# Isolation and Characterization of a Broad Spectrum Bacteriocin from* Bacillus amyloliquefaciens* RX7

**DOI:** 10.1155/2016/8521476

**Published:** 2016-04-28

**Authors:** Kong Boon Lim, Marilen P. Balolong, Sang Hoon Kim, Ju Kyoung Oh, Ji Yoon Lee, Dae-Kyung Kang

**Affiliations:** ^1^Department of Animal Resources Science, Dankook University, Cheonan 31116, Republic of Korea; ^2^National Instrumentation Center for Environmental Management, Seoul National University, Seoul 08826, Republic of Korea

## Abstract

We isolated a* Bacillus* strain, RX7, with inhibitory activity against* Listeria monocytogenes* from soil and identified it as* Bacillus amyloliquefaciens* based on 16S rRNA gene sequencing. The inhibitory activity was stable over a wide range of pH and was fully retained after 30 min at 80°C, after which it decreased gradually at higher temperatures. The activity was sensitive to the proteolytic action of *α*-chymotrypsin, proteinase-K, and trypsin, indicating its proteinaceous nature. This bacteriocin was active against a broad spectrum of bacteria and the fungus* Candida albicans*. Direct detection of antimicrobial activity on a sodium dodecyl sulfate-polyacrylamide gel suggested an apparent molecular mass of approximately 5 kDa. Ammonium sulfate precipitation and anion-exchange and gel permeation chromatography integrated with reverse phase-high-performance liquid chromatography were used for bacteriocin purification. Automated N-terminal Edman degradation of the purified RX7 bacteriocin recognized the first 15 amino acids as NH_2_-X-Ala-Trp-Tyr-Asp-Ile-Arg-Lys-Leu-Gly-Asn-Lys-Gly-Ala, where the letter X in the sequence indicates an unknown or nonstandard amino acid. Based on BLAST similarity search and multiple alignment analysis, the obtained partial sequence showed high homology with the two-peptide lantibiotic haloduracin (HalA1) from* Bacillus halodurans*, although at least two amino acids differed between the sequences. A time-kill study demonstrated a bactericidal mode of action of RX7 bacteriocin.

## 1. Introduction

Bacteriocins are ribosomally synthesized antimicrobial peptides which are secreted to act against closely related bacterial species without affecting the producing strain [1]. To address increasing bacterial resistance to conventional antibiotics, bacteriocins are now considered as alternative antimicrobials for the treatment of human (and possibly animal) infections [2]. Furthermore, since minimally processed foods with no chemical preservatives are in demand by consumers, research into natural antimicrobial agents such as bacteriocins [3] has been increasing. Lactic acid bacteria (LAB) bacteriocins are studied widely due to their potential use as biopreservatives in the food industry because many strains have been “generally recognized as safe” (GRAS) status [4]. The lantibiotic nisin, which contains unusual amino acids such as lanthionine and *β*-methyllanthionine, is the most studied bacteriocin to date and is the only bacteriocin currently used as a food additive [5]; however, the use of nisin is limited due to its very low activity at neutral or alkaline pH. Therefore, the search for novel bacteriocins with improved biochemical properties, including stability over a wide pH range, thermostability, and a broad antimicrobial spectrum, is of great interest for applications in foods.

Members of the* Bacillus* group are considered to be good producers of antimicrobial substances such as peptide and lipopeptide antibiotics, as well as bacteriocins [6]. Interestingly,* Bacillus* represents an alternative genus for the identification of bacteriocins because it includes many industrial species and has a history of safe use in the food industry [5]. It is also considered to be the second most important bacteriocin producer following LAB [3]. Therefore, the ability to screen for antimicrobial* Bacillus* strains is of major interest in bacteriocin research since this genus produces a diverse array of antimicrobial peptides [6, 7].

Several bacteriocins and bacteriocin-like inhibitory substances (BLIS) produced by* Bacillus amyloliquefaciens* have been described, most of which are inhibitory to Gram-positive bacteria, but lack activity against Gram-negative bacteria. Here, we identified and characterized a novel bacteriocin from* B. amyloliquefaciens* RX7. This bactericidal protein exhibits a broad antimicrobial spectrum, inhibiting several Gram-negative and Gram-positive bacteria.

## 2. Materials and Methods

### 2.1. Isolation of Antimicrobial Microorganisms

Soil samples were collected from a farm in Cheonan city, Korea. They were mixed with sterile water (1 : 10, w/v), homogenized, and heated at 50°C for 60 min in a water bath. One mL of this suspension was inoculated into 100 mL of tryptic soy broth (Difco, USA) and incubated at 50°C for 24 h, after which microbial growth was monitored based on changes in turbidity of the cultures. Aliquots of the cultures were inoculated onto TSB plates, incubated at 30°C, and single colonies were isolated and screened for antimicrobial activity. The antimicrobial activity was performed according to Ramachandran et al. [8] with modifications. It was expressed as the diameter of the inhibition zones around the wells using agar well diffusion assays with* Listeria monocytogenes* ATCC 19114 as the indicator strain. Other indicator strains were propagated in appropriate media as indicated in Table 1.

### 2.2. Antimicrobial Activity Assay

Antimicrobial activity was detected using the agar well diffusion assay and tested against all indicator strains (Table 1) grown in their respective media, as indicated (Difco, USA). An aliquot of culture was centrifugated at 6,000 g for 15 min at 4°C (Mega 21R, Hanil Co., Republic of Korea), filtered using 0.45 *μ*m pore-size cellulose acetate syringe filters (Advantec Co., Japan) to remove cells, then applied to wells on agar plates. Experiments were done in no less than five individual trials having no less than three replications.

The plates were incubated at the optimal temperature of the test organism. The bacteriocin titer was determined using the serial twofold dilution method. Activity was defined as the reciprocal of the dilution after the last serial dilution giving a zone of inhibition and was expressed as activity units (AU) per milliliter. All measurements were done in triplicate and the means are shown as the results.

### 2.3. Bacterial Identification

*Bacillus amyloliquefaciens* strain RX7 was identified taxonomically based on phenotypic and physiological characteristics using the Analytical Profile Index (API) test system (Biomérieux, France) and analysis of a partial 16S rDNA sequence. RX7 genomic DNA was prepared from an overnight culture using the phenol-chloroform extraction method. Amplification of the 16S rDNA gene by polymerase chain reaction (PCR) using the F1 (5′-AGAGTTTCCTGGCTCAG-3′) and R3 (5′-AAGGAGGTGATCCAGCC-3′) primers was performed under the following conditions: initial denaturation at 94°C for 5 min, 35 cycles of 95°C for 1 min, 55°C for 1 min, and 72°C for 90 s and a final extension at 72°C for 7 min. In addition, a* Bacillus subtilis*-specific primer set, ytcP-F (5′-GCTTACGGGTTATCCCGC-3′) and ytcP-R (5′-CCGACCCCATTTCAGACATATC-3′), was used to differentiate between* B. subtilis* and* B. amyloliquefaciens* under the following conditions: initial denaturation at 94°C for 5 min, 30 cycles of denaturation at 94°C for 30 s, annealing at 50°C for 1 min, and primer extension at 72°C for 1 min. Purified PCR fragments were sequenced with both primers and compared with 16S rRNA gene sequences in the public database using BLAST. API CHB50 bacterial identification analysis was also performed according to the manufacturer's instructions.

### 2.4. Purification of Bacteriocin from the RX7 Strain

After aerobic cultivation of* B. amyloliquefaciens* RX7 in 500 mL Erlenmeyer flasks containing 200 mL of TSB at 30°C for 6 h, cell-free supernatant (CFS) was obtained by centrifugation at 8,000 ×g for 20 min and for 15 min at 4°C (Hanil Science Industrial Co., Republic of Korea). The resulting supernatant was passed through a 0.45 *μ*m filter (Advantec Co., Japan). An ammonium sulfate precipitation at 60% saturation was performed in the culture filtrate. The resulting pellets were resuspended in 20 mM phosphate buffer (pH 7.0), followed by dialysis overnight (2-kDa cut-off, Sigma, USA). The dialyzed sample was applied to a Q Sepharose Fast-Flow resin anion-exchange column (GE Healthcare Life Sciences, USA) equilibrated with 20 mM phosphate buffer (pH 7) and eluted by a gradient of 2 M NaCl in the same buffer. Active fractions were collected, desalted, and concentrated using Sep-Pak C18 cartridges (Waters, USA). Active peptides were subsequently placed on a BioSep-SEC-S2000 gel permeation column (Phenomenex, USA) previously equilibrated with 50 mM phosphate buffer (pH 7.0) integrated in a HP1000 reverse phase-high-performance liquid chromatography (HPLC) system (Hewlett Packard, USA). The eluates were monitored based on their UV absorbance at 280 nm. Peptides were separated under isocratic conditions and subjected to N-terminal sequencing by Edman degradation.

### 2.5. Effects of pH, Temperature, Enzymes, and Organic Solvents on Antimicrobial Activity

To evaluate pH stability, the pH of crude bacteriocin solution was adjusted with 1 N HCl and 1 N NaOH and readjusted to pH 7 after incubation for 2 h at 37°C. To evaluate thermal stability, the crude bacteriocin solution was incubated at 4, 37, 50, 80, and 100°C for 15–30 minutes. All samples were cooled to room temperature before activity assays. To analyze sensitivity to various enzymes, the crude bacteriocin solution was treated at 37°C for 2 h with 10 mg/mL (final concentration) of the following enzymes: *α*-amylase, *α*-chymotrypsin, lipase, proteinase-K, pepsin, or trypsin. All enzymes used were from Sigma (USA) and Takara (Japan). The samples were then boiled for 2 min to denature the enzymes and cooled to room temperature. Samples were exposed to various organic solvents at a final concentration of 10% (v/v) to explore their effects on bacteriocin activity. After incubation for 2 h at 37°C, the residual activity was recorded. All treated samples were tested for residual activity against* L. monocytogenes* ATCC 19114, as described above.

### 2.6. Mode of Action

To determine the mode of action of the bacteriocin, the crude bacteriocin was added at a final concentration of 80 AU/mL to the culture at the mid-exponential growth phase of* L. monocytogenes* ATCC 19114 in 20 mL of TSB medium. Viable cells were counted at 1-hour intervals for the next 8 hours. Viable cell counting was repeated in triplicate and the mean values were calculated.

### 2.7. Molecular Weight Estimation

Antimicrobial activity was detected using tricine-sodium dodecyl sulfate-polyacrylamide gel electrophoresis (SDS-PAGE) as described previously [9]. Briefly, the crude bacteriocin sample (100 *μ*g) was loaded onto a 16.5% polyacrylamide gel and electrophoresis was performed at 100 V for 5 h. After electrophoresis, half of the gel was stained with Coomassie blue R-250 and the other half was washed with sterile water for 5 h with a water change every 30 min to remove SDS. The washed gel was overlaid with soft agar (0.7%, w/v) containing* L. monocytogenes* ATCC 19114 (OD600 0.4–0.8) cells. The overlaid gel was incubated for 24 h at 37°C and the clear zone of inhibition was measured. Molecular weight standards were obtained from Sigma (USA).

### 2.8. N-Terminal Sequencing

N-terminal amino acid sequencing of the active peak obtained from the HPLC analysis was performed by Edman degradation on a Procise 492 Protein Sequencing System [10, 11] (Applied Biosystems, USA).

## 3. Results

### 3.1. Isolation and Taxonomical Identification of a Bacteriocin-Producing Strain

A bacterial strain designated RX7, which exhibited antimicrobial activity against* L. monocytogenes*, was isolated from a soil sample. The RX7 strain was a short rod-shaped, Gram-positive bacterium (data not shown). Taxonomical analysis of the RX7 isolate using the API 50CHB bacterial identification system and homology analysis of the nucleotide sequence of the 16S rRNA gene identified the RX7 strain as* Bacillus subtilis* or* B. amyloliquefaciens*. Further examination using polymerase chain reaction (PCR) analysis showed the absence of the* B. subtilis*-specific* ytcP* gene encoding a hypothetical ABC-type transporter [12], which confirmed that RX7 was* B. amyloliquefaciens.* The producer strain was identified as* B. amyloliquefaciens* and the nucleotide sequence of the 16S rDNA of the RX7 strain was deposited in GenBank under the accession number KU301791.

### 3.2. Antimicrobial Spectrum

We tested the antimicrobial activity of a cell-free supernatant (CFS) against Gram-positive and Gram-negative bacteria (Table 1). The RX7 bacteriocin exhibited its highest activity against* L. monocytogenes*. It was also active against both Gram-positive pathogenic and spoilage bacteria such as* Bacillus cereus* and* Staphylococcus aureus*, and against pathogenic Gram-negative bacteria including* Escherichia coli*,* Pseudomonas aeruginosa*,* Salmonella enteritidis*, and* S. gallinarum*. RX7 bacteriocin also inhibited the fungus* Candida albicans*. These findings demonstrated the broad inhibitory spectrum of the bacteriocin produced by* B. amyloliquefaciens* RX7.

### 3.3. Effects of pH, Temperature, Enzymes, and Organic Solvents

Table 2 summarizes the effects of various treatments and conditions on the activity of purified RX7 bacteriocin against* L. monocytogenes* ATCC 19114. The antimicrobial activity was sensitive to *α*-chymotrypsin, proteinase-K, and trypsin, indicative of the proteinaceous nature of the antimicrobial substance. Full activity was still observed when exposed to 80°C for 30 minutes. Activity remained stable up to 100°C, but only 20% activity remained when subjected to 121°C for 15 min. Likewise, RX7 bacteriocin activity was stable in the presence of organic solvents and even under a wide range of pH conditions. Full activity was observed in the range of pH 3–8.

### 3.4. Mode of Action

**Figure 1:**
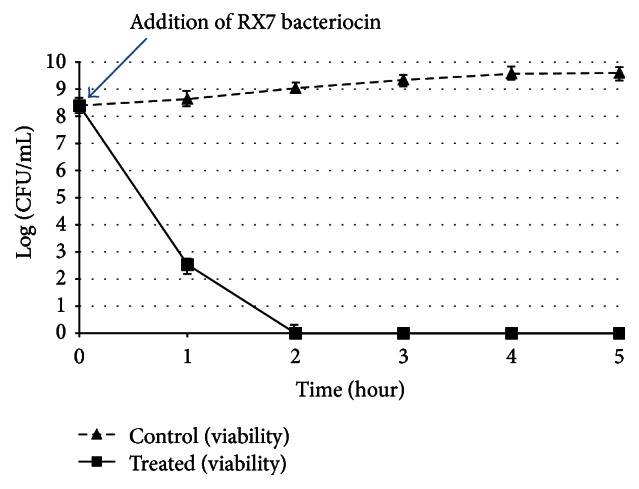
Bactericidal activity of RX7 bacteriocin against* Listeria monocytogenes* ATCC 19114. Viability of control (triangles) and treated (squares) cells was monitored. The time of RX7 bacteriocin addition is indicated by the arrow. Each point represents the mean of three independent experiments.

After addition of RX7 bacteriocin (80 AU/mL) to* L. monocytogenes* adjusted to 4.2 × 10^8^ colony forming units (CFU)/mL in TSB agar culture medium, the CFU were counted following various incubation periods (Figure 1). After the addition of RX7 bacteriocin, the population of cells decreased quickly, suggestive of a bactericidal mode of action. The incubation time for complete bactericidal action (i.e., no colony formation on TSB agar) was 2 h.

### 3.5. Molecular Weight Estimation and N-Terminal Amino Acid Determination

To estimate the molecular size of the bacteriocin, an ammonium sulfate precipitate of* B. amyloliquefaciens* RX7 culture supernatant was prepared as described in Section 2. The molecular size of RX7 bacteriocin was estimated based on tricine-sodium dodecyl sulfate-polyacrylamide gel electrophoresis (SDS-PAGE). The specific band associated with the antibacterial activity against* L. monocytogenes* was found at approximately 5 kDa, indicated by an observed single zone of inhibition from the sample after ammonium sulfate precipitation on the basis of its position (data not shown).

**Figure 2:**
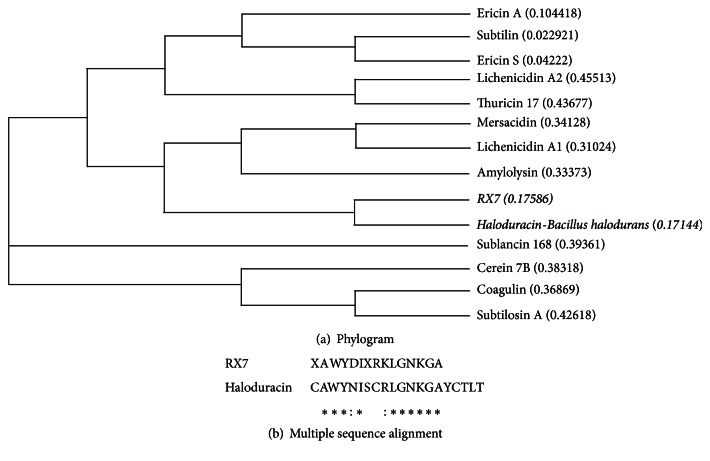
(a) Phylogram based on amino acid sequence homology of bacteriocins produced by* Bacillus* species. (b) Sequence alignment of RX7 bacteriocin and haloduracin A1 produced by* Bacillus halodurans* generated using MUSCLE 3.8 and Clustal 2.1 software.

Automated N-terminal Edman degradation of the purified RX7 bacteriocin recognized the first 15 amino acids as NH_2_-X-Ala-Trp-Tyr-Asp-Ile-Arg-Lys-Leu-Gly-Asn-Lys-Gly-Ala, where the letter X in the sequence represents an unknown or nonstandard amino acid. Based on BLAST similarity search and multiple alignment analysis, the obtained partial sequence showed high homology with the lantibiotic and haloduracin, from* Bacillus halodurans* [13]; however, at least two amino acids differed based on sequence comparison (Figure 2).

## 4. Discussion

Bacteriocins from* Bacillus* species, together with those from LAB, are gaining considerable attention for applications in human and animal health. We isolated the RX7 strain, which is active against the food pathogen* L. monocytogenes*, from a local soil sample and identified it as* B. amyloliquefaciens* based on biochemical profiling and 16S rDNA sequencing. DNA-based identification methods such as 16S rRNA gene sequencing have been used widely for the purpose of identification and typing of microorganisms isolated from natural environments [12]. However, identification based on rRNA gene sequences sometimes fails to distinguish one species from the other if they share highly similar rRNA genes, as in the case of* B. subtilis* and* B. amyloliquefaciens*. In this work, a primer pair specific for* ytcP* gene, which* B. subtilis* contains and* B. amyloliquefaciens* does not, was used to distinguish these species. However, further confirmation experiments are needed in the future.

The sensitivity of the antimicrobial substance from* B. amyloliquefaciens* RX7 to proteases was indicative of its proteinaceous nature, leading us to classify it as a bacteriocin. This compound exhibited broad spectrum antibacterial activity. In addition, RX7 bacteriocin inhibits the growth of various species of Gram-positive bacteria, including* L. monocytogenes*,* B. cereus*, and* S. aureus*, and Gram-negative bacteria, including* E. coli*,* P. aeruginosa*,* S. enteritidis*, and* S. gallinarum*. This characteristic is atypical of bacteriocins in that bacteriocins produced by Gram-positive bacteria are mostly inhibitory towards Gram-positive bacteria and are less effective against Gram-negative bacteria [14].

To date, several bacteriocin or bacteriocin-like inhibitory substances (BLIS) from* B. amyloliquefaciens* have been reported: BLIS RC-2, which is antagonistic to bacterial and fungal plant pathogens [15]; BLIS 5006, which is antagonistic to food-spoilage bacteria with declining activity at alkaline pH [16]; and BLIS 5940, which is active against* Clostridium perfringens* [17]. Other well-characterized bacteriocins from* B. amyloliquefaciens* are amylolysin [18] and amylocyclicin [19], which are primarily active against Gram-positive bacteria including food pathogenic* Listeria*. Subtilosin A, a posttranslationally modified peptide (Class I) produced by* B. subtilis* and* B. amyloliquefaciens*, has antimicrobial activity against Gram-positive and Gram-negative bacteria [20].

However, N-terminal sequencing and sequence alignment of the bacteriocin from RX7 showed no homology to subtilosin A, but significant similarity with haloduracin A1, a lantibiotic, which is a posttranslationally modified peptide (Class I) produced by* B. halodurans* [13]. In addition, the estimated molecular size of RX7 bacteriocin and its stability at high temperature and in solvents are similar to those of haloduracin. However, at least two amino acids were different based on sequence comparison. Moreover, haloduracin inhibits only Gram-positive bacteria and is alkaliphilic in nature unlike RX7 bacteriocin, indicating that RX7 bacteriocin from* B. amyloliquefaciens* differs from the lantibiotic haloduracin.

Recently, sonorensin was isolated from a marine isolate of* Bacillus sonorensis* and it exhibited broad-spectrum antibacterial activity towards both Gram-positive and Gram-negative bacteria [21]. Sonorensin had significant similarity to the putative thiazole-containing heterocyclic bacteriocin of* Bacillus licheniformis* ATCC 14580 (accession number YP_006712426.1), and its N-terminal sequence is homologous to the reader sequences of protoxins from various* Bacillus* strains. This suggested that sonorensin belongs to a new subfamily of bacteriocin, heterocycloanthracin, a group of putative peptides containing oxazole and/or thiazole heterocycles [22].

RX7 bacteriocin is similar to subtilosin A in that it has broad antimicrobial activity, thermostability, and antimicrobial activity under a broad range of pH conditions. However, the two bacteriocins exhibit amino acid sequence differences; RX7 bacteriocin is similar to haloduracin in the N-terminal sequences. The characteristics of RX7 bacteriocin are in common with Class I bacteriocins (lantibiotics). Further study to identify gene clusters involved in the biosynthesis and mechanism of RX7 bacteriocin is required for structural characterization.

Here, we identified and characterized a potentially novel bacteriocin produced by a strain of* B. amyloliquefaciens*. With its broad spectrum inhibitory properties, thermostability, and tolerance to a broad range of pH conditions and various solvents, it may be applicable to food preservation and human and animal health.

## Figures and Tables

**Table 1:** Antimicrobial spectra of the RX7 bacteriocin.

Indicator organism	Media	Inhibition
		zone (mm)^*∗*^
Gram-positive		
*Bacillus cereus *KCTC 1661	LB	++
*Bacillus licheniformis *KCCM 12145	NB	++
*Enterococcus faecalis *(VRE) CCARM 0011	NB	++
*Listeria monocytogenes *ATCC 19114	TSB	+++
*Streptococcus agalactiae *ATCC 13813	TSB	++
*Staphylococcus aureus* KFRI 00188	NB	++
*Staphylococcus aureus *subsp.* aureus* (MRSA) KCCM 40510	NB	+
*Streptococcus mutans *ATCC 25175	BHI	+
*Lactobacillus acidophilus *KCCM 32820	MRS	+
*Lactobacillus delbrueckii* KCCM 11357	MRS	+
*Lactobacillus johnsonii* PF01 (our isolate)	MRS	+
*Lactobacillus salivarius* CPM7 (our isolate)	MRS	+
*Lactobacillus plantarum* KCCM 11322	MRS	+

Gram-negative		
*Escherichia coli* K88	LB	++
*Klebsiella pneumoniae *subsp.* pneumoniae *KCCM 11418	NB	+
*Pseudomonas aeruginosa* KCCM 11266	NB	++
*Pseudomonas aeruginosa* CCARM 2003	LB	++
*Shigella flexneri *KCCM 40414	NB	++
*Salmonella enteritidis *KCCM 12021	NB	++
*Salmonella enteritidis* KVCC-BA0700654	NB	++
*Salmonella gallinarum *KVCC-BA0700722	NB	++
*Salmonella pullorum* KVCC-BA0702509	NB	+
*Salmonella typhimurium* KCCM 40253	NB	++

Fungi		
*Candida albicans *KCTC 7122	PDA	++

ATCC: American Type Culture Collection; CCARM: Culture Collection of Antimicrobial Resistant Microbes; KFRI: Kerala Forest Research Institute; KCCM: Korean Culture Center of Microorganisms; KCTC: Korean Collection for Type Culture.

TSB: tryptic soy broth; NB: nutrient broth; LB: Luria-bertani; BHI: brain heart infusion; MRS: de Man Rogosa and Sharpe.

^*∗*^Activity is expressed as the diameter of the inhibition zone around the well: +, less than 10 mm; ++, less than 20 mm; +++, less than 30 mm.

**Table 2:** Effects of enzymes, temperature, pH, and organic solvents on the antimicrobial activity of RX7 bacteriocin.

Treatment	Residual activity (%)^*∗*^
None (control)	100
Enzymes^†^	
*α*-Amylase	100
*α*-Chymotrypsin	0
Lipase	100
Proteinase-K	0
Pepsin	100
Trypsin	0
Heat	
4°C/30 min	100
37°C/30 min	100
50°C/30 min	100
80°C/30 min	100
100°C/30 min	80
121°C/15 min	20
pH	
1	80
2	90
3	100
4	100
5	100
6	100
7	100
8	100
9	90
10	80
Organic solvents^‡^	
Acetone	90
Acetonitrile	90
Butanol	80
Chloroform	80
Ethanol	90
Methanol	80

^*∗*^Residual activity compared with antimicrobial activity before the treatment.

^†^Enzyme concentrations were 10 mg/mL.

^‡^10% concentration.
